# A Foreseeable Tissue Engineering Approach to Overcome the Neurogenic Bladder-Related Detrusor/Urethral Rhabdosphincter Dyssynergia

**DOI:** 10.3389/fped.2014.00122

**Published:** 2014-11-10

**Authors:** Contardo Alberti

**Affiliations:** ^1^LD of Surgical Semeiotics, University of Parma, Parma, Italy

**Keywords:** neurogenic bladder, detrusor-sphincter dyssynergia, tissue engineering, pediatrics, urology

I have read, with high interest, the Jednak’s review article “The evolution of bladder augmentation: from creating reservoir to reconstituting an organ” (1), where the literature regarding this subject has been carefully taken into consideration. Particularly, about the tissue engineering-based augmentation cystoplasty, the attention has been focused on the results reached by Atala and his group in patients suffering from end-stage myelomeningocele-induced poorly compliant/high pressure bladder (2).

Nevertheless, such tissue engineered arrangement remains functionally conditioned by spinal cord neuropathy-due detrusor/urethral rhabdosphincter dyssynergia. Hence, in my opinion, it would be suitable, for these patients, to implant, after total cystectomy together with removal of the urethral rhabdosphincter, a tissue engineered neobladder–rhabdosphincter complex – quite not influenced by spinal cord neuropathy effects – provided with inside neobladder wall embedded tension micro-electro-sensors (correlatively to intra-neobladder pressure) with micro-loop antenna to send, beyond a properly adjustable wall tension value threshold, modulated wireless e-m signals toward a rhabdosphincterial receiver–converter micro-electro device to promote, in turn, by suitable e-m field generation, the rhabdosphincter relaxation simultaneously with the neobladder contraction. What should be quite reversible following the micturition-due intra-neobladder pressure drop below the arranged levels.

Bright advances in the scaffold fabrication, emerging from recent progress in the field of both nanotechnology and material science research – from different “smart” synthetic polymers to silk fibroin-based biomaterials – besides in stem-cell biology, could make feasible a suitable setting of micro-electro-sensors inside the mentioned bladder/rhabdosphincter tissue engineered complex (3–5).

## Conflict of Interest Statement

The author declares that the research was conducted in the absence of any commercial or financial relationships that could be construed as a potential conflict of interest.
